# Epidemiological Characteristics and Maternal Risk Factors of Microtia and Aural Atresia in Kazakhstan

**DOI:** 10.1055/s-0044-1792015

**Published:** 2025-01-27

**Authors:** Assel Imangaliyeva, Rimma Suatbayeva, Tatyana Slazhneva, Aigul Medeulova, Zhanetta Mukanova, Amangeldy Kulimbetov, Neilya Mileshina, Natalya Glushkova, Marina Izmailovich, Yuliya Semenova

**Affiliations:** 1 Higher School of Public Health, Kazakhstan Medical University, Almaty, Kazakhstan; 2 Department of Non-Communicable Disease Prevention, National Centre for Public Health, Ministry of Healthcare of the Republic of Kazakhstan, Astana, Kazakhstan; 3 Department of Otorhinolaryngology, Kazakh National Medical University named after S. D. Asfendiyarov, Almaty, Kazakhstan; 4 Department for Prevention and Correction of the Hearing Disorders, National Research Centre for Audiology and Hearing Rehabilitation, Moscow, Russia; 5 Department of Epidemiology, Biostatistics and Evidence-Based Medicine, Al-Farabi Kazakh National University, Almaty, Kazakhstan; 6 Department of Internal Diseases, Karaganda Medical University, Karaganda, Kazakhstan; 7 School of Medicine, Nazarbayev University, Astana, Kazakhstan

**Keywords:** microtia, children, frequency, risk factors, meatal atresia

## Abstract

**Introduction** Microtia and aural atresia present congenital ear anomalies that affect external ear and are associated with conductive hearing loss. Both anomalies result from exposure to various prenatal risk factors, most common during the first trimester of pregnancy.

**Objective** This study was aimed at epidemiological analysis of microtia/atresia and associated risk factors in the Kazakhstani population.

**Methods** A retrospective study in two stages. First, a cross-sectional analysis of microtia/ atresia frequencies from 2015 to 2019 on the basis of official statistics obtained from the Republican Centre for E-Health. Then, a case-control study was carried out to elucidate maternal risk factors associated with occurrence of microtia/atresia. We recruited patients presented in Almaty, Kazakhstan, between September 2021 and February 2022.

**Results** There was a substantial regional variation in the rates of both aural atresia and microtia/anotia. Mothers of children with microtia disclosed toxoplasmosis, other agents (including HIV, syphilis, varicella), rubella, cytomegalovirus, herpes simplex (TORCH) infections during pregnancy more often than those of healthy children (45.8 versus 7.3%; *p* < 0.001). Exposure to different chemicals during pregnancy was mentioned more frequently by mothers of children with microtia when compared with the healthy controls (18.1 versus 8.1%; *p* = 0.035). Self-reporting of alcohol consumption and intake of antibiotics was also significantly higher in mothers of children with microtia (31.9 and 36.1% respectively).

**Conclusion** Elucidation of microtia/atresia epidemiology is important due to their imposed social and economic burden, associated with treatment and rehabilitation costs.

## Introduction

Microtia and aural atresia are congenital ear anomalies that affect the external ear and are associated with conductive hearing loss. In microtia, the outer ear is deformed and smaller than normal size, while in atresia external auditory canal is absent. Both auditory anomalies result from exposure to various prenatal risk factors, which occur during the first trimester of pregnancy. These conditions may coincide and be unilateral or bilateral. In case of unilateral defects, the right ear is more commonly affected than the left. Hearing loss is typically present in both, being more profound in atresia. A spectrum of other facial anomalies might occur along with microtia and atresia, further complicating the child's health status. 1

There are several grading systems of microtia, but the Marx classification is universally used. According to this classification, grade I is a slightly smaller auricle with presence of all subunits, while in grade II the subunits are severely underdeveloped or even absent. The so-called “peanut ear” is the most common manifestation of grade III microtia, characterized by the anterosuperior rotation of the lobule. Anotia is defined as the complete absence of the auricle and is classified as Grade IV microtia. 2 The Jahrsdoerfer grading scale is used to evaluate the patients' candidacy for atresia repair based on presence or absence of nine anatomical structures of the ear. Higher scores indicate better prognosis for postoperative hearing. 3

A number of maternal risk factors were attributed to the development of microtia and atresia, among which are preexisting disease or acute illness during the first trimester of pregnancy, high parity and multiple births, low level of schooling, advanced age, use of certain medications, alcohol intake, and exposure to high altitude. 4 5 Also, a set of neonatal risk factors were reported, including male gender, low birth weight, and belonging to certain ethnic groups. The paternal risk factors are relatively poor studied and so far, only increased age was attributed to microtia and atresia. 4 5 Major mutations in single genes could also predispose to their development, and more than 20 syndromes were linked to this disorder, such as the Treacher-Collins syndrome with a mutation in the TCOF1 gene. 6

The prevalence of microtia ranges between 0.83 and 4.34 per 10 thousand births. 6 In half of the cases the disease is isolated, and only one out of ten cases is bilateral. 7 According to the systematic review, the global prevalence of microtia/anotia is 2.06, with a confidence interval (CI) of 2.02 to 2.10, and the rates are higher in Americas, Northern Europe and Asia. 8 The global prevalence of aural atresia was estimated to be 1 per 10 thousand births and certain populations, like Hispanics, have higher rates. 9 There is a lack of studies published within the past decade on epidemiology of microtia and aural atresia in different world nations. The present work aimed for an epidemiological analysis of microtia/atresia and associated risk factors in the Kazakhstani population.

## Methods

### Study on the Frequency of Microtia and Atresia

To evaluate the frequency of microtia and aural atresia, we obtained official statistics from the Republican Center for E-Health, Ministry of Healthcare of the Republic of Kazakhstan for a 5-year period (2015–2019). The center maintains the electronic patient registry, which comprises health records of all patients presented at different healthcare establishments of the country, including primary, secondary and tertiary levels of care. The electronic patient registry was set up in 2015 and serves as the centralized database that provides relevant statistical information on the basis of diagnostic codes, as specified by the 10th Revision of the International Classification of Diseases (ICD-10). We obtained medical records of patients with the following ICD-10 codes: Q16.0, congenital absence of (ear) auricle; Q16.1, congenital absence, atresia and stricture of the external auditory canal; and Q17.2, microtia. More information on how electronic patient registry operates could be obtained elsewhere. 10

Incidence and prevalence rates were calculated per 100 thousand of child population. Data on the overall number of children, aged 0 to 14 years, were extracted from the annual statistical compilation issued by the Agency of Statistics of Kazakhstan. Prevalence of microtia and atresia was calculated based on the following formula:

*Number of all cases registered within 1 year among children aged 0 to14 years / midyear number of children aged 0 to 14 years × 100,000.*

The period prevalence was calculated as follows:

*Number of all cases registered from 2015 to 2019 among children aged 0 to 14 years / number of children aged 0 to 14 years over the same time period × 100,000*

Meanwhile, the following formula was applied for calculation of microtia and atresia incidence rates:

*Number of new cases registered within 1 year among children aged 0 to 14 years / midyear number of children aged 0 to 14 years × 100,000.*

### Study on Maternal Risk Factors for Microtia and Atresia

To elucidate the maternal risk factors associated with development of microtia and atresia, we conducted a case-control study, which involved patients presented in Almaty, Kazakhstan, between September 2021 and February 2022. This hospital serves as the referral institution for the treatment of microtia and aural atresia in Kazakhstan. We enrolled only those patients, who had no signs of syndromic microtia, including such congenital anomalies, as renal, cardiac, and limb defects. We also excluded patients with a family history of microtia and atresia.

The diagnosis of microtia and atresia was made by an experienced otorhinolaryngologist on the basis of detailed physical examination. Marx classification was used to classify microtia by grade and since the earlier epidemiological study showed that severe microtia is more likely to be associated with maternal risk factors, 11 we included only patients with microtia grades III-IV accompanied by aural atresia. Overall, we enrolled 71 microtia/atresia patients, who were matched 1:1.7 with normal controls, adjusted by age and sex. The mean ± standard deviation (SD) age for the case-control study was 4.8 ± 3.3 years, with the age range being from 6 months to 13 years. The controls were identified via field investigation and comprised healthy children attending educational establishments adjacent to the Hospital or those who were admitted for other reasons, apart from microtia and any syndrome. The controls were also examined by an experienced otorhinolaryngologist to ascertain the absence of any congenital auricular malformation. Both cases and controls could be considered representative of the Kazakhstani population, as Almaty, the largest city in the country, has a demographic composition similar to the overall Kazakhstani population, characterized by diverse ethnicities and socioeconomic backgrounds.

To interview mothers, we constructed a questionnaire on the basis of a careful revision of available scientific reports on risk factors for microtia and atresia, which potentially occur during the first trimester of pregnancy. 5 The resulting questionnaire covered both potential risk factors and children's individual characteristics. We divided the questionnaire into the following sections: sociodemographic profile, maternal health and obstetric history, maternal habits and exposures. The section of sociodemographic profile covered questions on children's age, gender, place of residence, maternal and paternal level of schooling. The section of maternal health and obstetric history included information on maternal age; history of miscarriage and threatened abortion; presence of certain disorders during pregnancy, like arterial hypertension, diabetes mellitus, hypercholesterolemia and some infections; hyperemesis gravidarum; and medication history. The infections covered included acute respiratory illness and toxoplasmosis, other agents (including HIV, syphilis, varicella), rubella, cytomegalovirus, herpes simplex (TORCH), as all pregnant women in Kazakhstan are tested for the above listed infections. The maternal risk factor “chemicals” was assessed based on whether the mother worked in the manufacturing of varnishes, paints, fertilizers, medicines, and chemicals before or during pregnancy.

### Ethics Statement

The present study was approved by the Local Ethics committee (extract from the protocol N°150 dated 05.08.2021). When collecting personal information of patients, the principles depictured in the Declaration of Helsinki were strictly followed and written informed consent was obtained from one of the parents.

### Statistical Analysis

All statistical analyses were performed using IBM SPSS Statistics for Windows (IBM Corp., Armonk, NY, USA) software, version 20.0. Before any statistical test was used, we tested the mode of data distribution by means of the Shapiro-Wilk test. Significance level of α < 0.05 was used. All quantitative data were reported as absolute numbers with their percentages and the Pearson chi-squared test was used to test the difference between study groups.

## Results

### Study on Microtia and Atresia Frequency

**Fig. 1:**
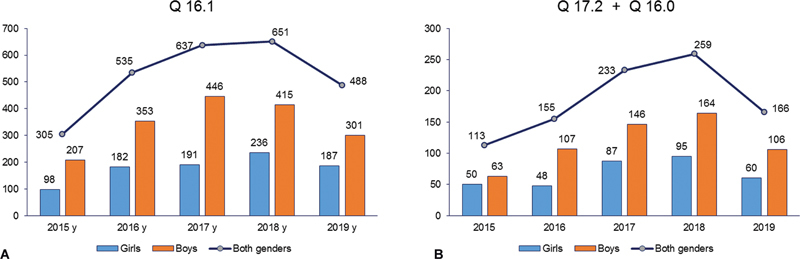
( **A** ) Number of children registered in the country with congenital absence, atresia, and structure of the external auditory canal (Q16.1). ( **B** ) Number of children registered in the country with congenital absence of ear (auricle, Q 16.0) and microtia (Q17.2).

The number of children followed because of congenital absence, atresia and stricture of external auditory canal (Q16.1) was bigger than the number of children registered with microtia (Q16.0) and congenital absence of ear (Q17.2). In general, there were more boys than girls and male to female ratio was about 2:1 for aural atresia and 1.7:1 for microtia and anotia ( Fig. 1 ).

**Fig. 2:**
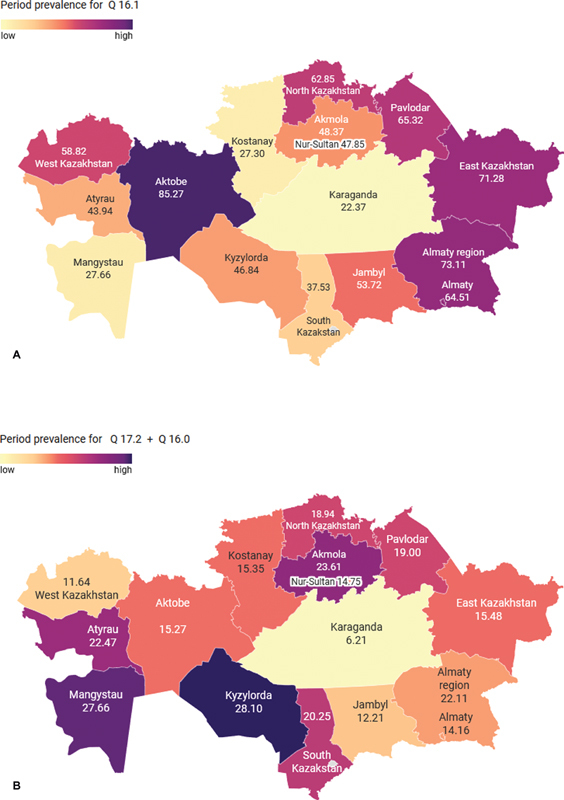
( **A** ) The regional variation in period prevalence of congenital absence, atresia and stricture of external auditory canal (Q16.1). ( **B** ) The regional variation in period prevalence of congenital absence of ear (auricle, Q 16.0) and microtia (Q17.2).

As shown in Fig. 2 , there was a significant regional variation in the period prevalence of both aural atresia and microtia/anotia. The lowest rates of atresia (22.37) and microtia/anotia (6.21) were seen in the Karaganda region. Meanwhile, the highest rate of aural atresia was observed in Aktobe region (85.27) and that of microtia/anotia was registered in Kyzylorda region (28.10). The national incidence of aural atresia ranged from 1.76 (2015) to 3.42 (2018), while the rates of microtia/anotia ranged from 0.68 (2015) to 1.37 (2017). Similar to the period prevalence, there was a substantial variation in the regional rates ( Table 1 ).

**Table 1:** Incidence of aural atresia (ICD-10 code Q16.1) and microtia/anotia (ICD-10 codes Q 17.2 and 16.0) across regions of Kazakhstan from 2015 to 2019

Region/city	Year									
	2015		2016		2017		2018		2019	
	Q 16.1	Q 17.2 + Q16.0	Q 16.1	Q 17.2 + Q16.0	Q 16.1	Q 17.2 + Q16.0	Q 16.1	Q 17.2 + Q16.0	Q 16.1	Q 17.2 + Q16.0
Akmola	0.59	0.59	2.33	0.00	2.88	2.88	2.84	2.27	2.26	1.70
Aktobe	4.59	0.00	5.73	0.00	2.96	0.42	2.86	0.00	0.79	1.98
Almaty region	1.98	1.08	4.14	1.21	3.96	0.99	3.20	0.80	3.44	0.62
Atyrau	2.17	0.54	4.67	0.00	2.49	1.99	1.92	0.48	1.40	0.00
East Kazakhstan	2.66	0.66	3.26	0.98	4.50	1.61	3.18	0.64	2.22	0.63
Jambyl	1.98	0.85	3.57	0.27	2.70	0.81	1.60	1.07	1.58	1.05
West Kazakhstan	1.30	0.65	5.04	0.00	3.67	0.00	4.17	0.60	4.07	0.58
Karaganda	0.64	0.32	0.63	0.00	1.24	0.00	2.15	0.31	1.83	0.61
Kostanay	0.00	1.72	2.28	1.14	1.70	1.70	1.13	1.70	2.27	1.14
Kyzylorda	1.64	1.64	4.00	1.60	3.51	2.34	5.33	3.81	2.99	2.24
Mangystau	0.98	0.00	0.47	0.93	2.23	4.01	4.70	3.85	2.05	1.23
Pavlodar	2.45	0.61	5.40	0.60	5.32	1.77	4.10	0.00	1.16	0.00
North Kazakhstan	0.00	0.00	3.43	1.72	1.72	0.00	2.58	0.00	1.74	0.00
South Kazakhstan	1.52	0.51	2.46	0.99	2.50	1.25				
Turkestan							1.34	2.02	2.12	1.06
Shymkent city							4.60	1.53	3.80	0.88
Almaty city	2.87	0.57	4.63	1.36	5.95	1.81	7.35	1.22	7.89	0.70
Nur-Sultan city	1.33	0.88	1.99	0.79	5.35	1.43	6.25	1.65	3.67	0.31
Kazakhstan republic	1.76	0.68	3.24	0.80	3.32	1.37	3.42	1.35	2.88	0.88

Abbreviation: ICD-10, International Classification of Diseases, 10th Revision.

### Study on Maternal Risk Factors for Microtia and Atresia

Table 2 presents the basic characteristics of patients with microtia/aural atresia. Many patients (62.0%) resided in different regions of Kazakhstan, while the rest were from the metropolis (Almaty city). The mean age of patients was 5 years old and more than half of them (66.2%) were males. The bulk of mothers (57.7%) had higher education, while almost equal numbers of fathers had secondary vocational and higher education (25 versus 26). There were more grade IV microtia patients (85.9%) and the disorder was right sided in 43.7% of cases.

**Table 2:** Characteristics of patients with microtia and aural atresia (n = 71)

Characteristics		n	%
Place of residence	Regional	44	62.0
	Metropolis	27	38.0
Age, years (mean ± SD)		5.0 ± 3.3	
Gender	Female	24	33.8
	Male	47	66.2
Maternal level of schooling	Secondary	16	22.2
	Secondary vocational	14	19.4
	Higher	41	57.7
Paternal level of schooling	Secondary	20	28.2
	Secondary vocational	25	34.7
	Higher	26	36.1
Severity of microtia	Grade III	10	13.9
	Grade IV	61	85.9
Laterality	Bilateral	21	29.6
	Unilateral Right side Left side	31 19	43.7 26.8

**Abbreviation:** SD, standard deviation.

The selected maternal risk factors for microtia and aural atresia are presented in Table 3 . There was a significant difference in maternal age between the study groups as mothers of healthy children were younger. Mothers of children with microtia disclosed TORCH infections during pregnancy more often than mothers of healthy children (45.8% versus 7.3%; *p* < 0.001). Also, personal history of gestosis differed significantly between the groups and was reported by 36.1% of mothers of children with microtia/aural atresia and just by 14.6% of mothers of healthy children. Exposure to different chemicals during pregnancy (varnishes, paints, fertilizers, class A chemicals) was mentioned more frequently by mothers of children with microtia as compared with the healthy controls (18.1% versus 8.1%; *p* = 0.035). Self-reporting of alcohol consumption and intake of antibiotics was also significantly higher in mothers of children with microtia (31.9 and 36.1% respectively).

**Table 3:** Selected maternal risk factors of microtia and aural atresia

Variables		Group				Test of difference	
		Controls (n = 123)		Cases (n = 71)		χ ^2^	*p* -value
		n	%	n	%		
Maternal disorders	Arterial hypertension	21	17.1	20	27.8	3.134	0.077
	Hypercholesterolemia	26	21.1	20	27.8	1.230	0.267
	Diabetes mellitus	24	19.5	16	22.2	0.251	0.616
Maternal age, years	≤ 30	66	53.7	21	29.2	11.062	0.004
	31–40	45	36.6	41	56.9		
	≥ 41	12	9.8	10	13.9		
History of disorders during pregnancy	Acute respiratory infection	30	24.4	18	25.0	0.009	0.924
	TORCH	9	7.3	33	45.8	39.868	< 0.001
	Gestosis	18	14.6	26	36.1	23.795	< 0.001
	Fatigue	51	41.5	34	47.2	0.613	0.434
Exposures during pregnancy	Chemicals	10	8.1	13	18.1	4.464	0.035
	Smocking	37	30.1	31	43.7	3.647	0.056
	Alcohol	21	17.1	23	31.9	6.026	0.014
Medication intake	Antibiotics	12	9.8	26	36.1	20.106	< 0.001
	Hormones	28	22.8	15	20.8	0.070	0.791
	Other medications	14	11.4	6	8.3	0.418	0.518
Past history of miscarriage and threatened abortion		12	9.8	15	20.8	3.2654	0.071

**Abbreviations:** χ ^2^ , Chi-squared; TORCH, toxoplasmosis, other agents (including HIV, syphilis, varicella, and fifth disease), rubella, cytomegalovirus, herpes simplex.

## Discussion

Elucidation of microtia/atresia epidemiology is important due to the imposed social and economic burden associated with treatment and rehabilitation costs and, in some instances, stigmatization. Better understanding of the underlying risk factors leads to the formulation of better prevention programs. Knowledge of the geographical distribution of patients helps to establish quality healthcare services where they are most needed. Availability and accessibility of health care services for congenital anomalies have another important dimension in Kazakhstani reality, as this can minimize the risk of child abandonment. 12

### Study on the Frequency Rates

There is high geographic variation in frequency rates of microtia and atresia across different world regions and ethnic groups. In general, the Hispanic population tends to have the highest microtia prevalence, 4 followed by Asian and Pacific populations. 8 Out of European states, high microtia prevalence was reported in Finland, which was very close to that seen in Latin American population. 13 Several explanations to high microtia rates in that last population exist, and living at high altitudes (above 2,500 meters) is among them. However, it is not clear if this phenomenon could be attributed to a high altitude alone or if it is due to a big prevalence of Native Americans among the highlanders. 6 In general, certain ethnicities are considered as a risk factor for microtia and studies performed on the United States population showed that the highest rates are observed among people of Asian, Pacific, and Hispanic ancestries. 14

However, the extent to which variability in observed rates could be explained by the differences in study design remains unclear. Most of studies reporting microtia rates are registry-based, which may present a source of bias as case ascertainment varies between different registries and more mild cases can be misinterpreted, particularly in the presence of more severe co-existing pathology. The findings of population-based studies may also be incomparable because of differences in research methodology and inclusion criteria used. This was a registry-based study, which comprised medical records of all patients presented both at out- and in-patient healthcare facilities across the country, which could be considered as one of the study's strengths. Still, the data lack disaggregation in terms of ethnicity, as Kazakhstani population is ethnically heterogeneous. 10 Another limitation comes from the coding's uncertain accuracy, given it was used by medical professionals at the time of data entry, as less severe cases of microtia may be underreported.

### Study on Maternal Risk Factors

Currently, there is no consensus regarding the impact of maternal age on microtia and atresia development. While some studies indicated that frequency of congenital anomalies of the outer ear increase with maternal age, 5 15 others failed to establish such relationship. 16 In general, maternal age is likely to confound the relationship between a previous history of miscarriage and microtia; 5 15 interestingly, a number of publications also attributed increased paternal age to a higher incidence. 5 17 Advanced maternal and paternal ages are also interrelated, since older women tend to have children with older men.

Apart from genetic predisposition, maternal disease and exposure to various environmental factors play a role in microtia and atresia development. As such, a number of studies reported that threat of abortion during the first trimester and history of miscarriage are related to the occurrence of microtia; 5 18 Nevertheless, it is worth noting that both could be provoked by different maternal factors, including infections, chronic diseases, and unhealthy lifestyles and, thus, these factors could be considered as confounders for the development of microtia. Self-reporting of threat of abortion during the first trimester of pregnancy and past history of miscarriage did not differ significantly between the cases and controls in this study, which could be explained by the relatively small sample size.

A range of viruses may act as teratogens due to their ability to impair the development of the first and second pharyngeal arches, causing congenital anomalies of the middle and outer ear. These same properties were attributed to retinoic acid administered at 2 to 5 weeks of gestation and some other medications. 19 Mothers of children with microtia more frequently reported antibiotic intake, while the use of other medications during pregnancy was not significantly different between cases and controls in this study. Certain maternal pathologies, like gestosis and anemia, were also reported as the risk factors for microtia, although the associated mechanisms were not identified. 20 In our study, mothers of children with microtia more often reported gestosis, ARI (Acute Respiratory Infection), and TORCH during pregnancy, although it is not clear to which extent this could be attributed to recall bias.

Exposure to smoking and alcohol intake were listed among the causes for microtia. 21 Both factors can impair cell migration and/or neural differentiation of the neural crest, which results in various structural defects, including congenital ear anomalies. 22 The present study found out that maternal alcohol consumption was associated with microtia/atresia, but the same was not true for smoking. In general, it is uncommon for women in Kazakhstan to smoke or consume alcohol during pregnancy. Therefore, our findings should be interpreted in light of this fact. However, there is a lack of epidemiological studies specifically focused on smoking and alcohol consumption rates among pregnant women in Kazakhstan. The available estimates only reflect the rates in the general population, which show lower consumption rates among Kazakhstani women compared to men. According to data from the World Health Organization (WHO), men aged 15 years and older consume 25 liters of pure alcohol per capita, while women consume 8.9 liters. 23 Similarly, the latest epidemiological evidence indicates that 38.5% of Kazakhstani men are current smokers, compared to 10.1% women. 24

Another limitation of our study arises from the fact that, in the maternal interviews, information on specific chemicals was not collected. This could contribute to recall bias related to chemical exposures between case and control mothers. Moreover, the genuine occurrence can be delineated as the quotient of the number of cases within a specified geographic area and temporal interval divided by the number of susceptible conceptions, a figure that remains largely uncertain. Additionally, unaddressed in the manuscript is the restriction of considering solely live births in the numerator.

Unlike microtia, maternal risk factors associated with the development of congenital aural atresia are rather poorly studied partly because these conditions often coexist. From this point of view, it would be logical to conclude that factors related to microtia can also provoke aural atresia. Although the latter is generally considered a multifactorial disorder, several risk factors were identified, which include maternal exposure to isotretinoin, thalidomide, and cocaine, as well as associated maternal diabetes. 25

To this date, the studies on maternal risk factors for microtia/aural atresia are rare, which is one of the advantages of our study. Another strength comes from the fact that all children were examined by an experienced otorhinolaryngologist, enabling proper ascertainment of microtia and atresia cases. Furthermore, we randomly recruited controls with adjustment by age and sex to minimize the effect of confounders and the recall bias associated with self-reporting. Still, because this was a case-control study, we could not avoid all limitations connected with this fact, including the need to rely on mothers' memories, which made the referral bias inevitable.

## Conclusion

Elucidation of microtia/atresia epidemiology is important, mainly due to the imposed social and economic burden associated with treatment and rehabilitation costs.
